# Investigation of the Influence of Manufacturing on Filament Production and Its Impact on Additive Manufactured Structures

**DOI:** 10.3390/polym17050651

**Published:** 2025-02-28

**Authors:** Mohamed Refat, Robert Maertens, Patrick Weiss, Frank Henning, Volker Schulze, Wilfried V. Liebig

**Affiliations:** 1 Institute for Applied Materials—Materials Science and Engineering, Karlsruhe Institute of Technology (KIT), Engelbert-Arnold-Str. 4, 73161 Karlsruhe, Germany; wilfried.liebig@kit.edu; 2 Fraunhofer Institute for Chemical Technology ICT, Joseph-von-Fraunhofer-Str. 7, 76327 Pfinztal, Germany; robert.maertens@ict.fraunhofer.de (R.M.); patrick.weiss@ict.fraunhofer.de (P.W.); frank.henning@ict.fraunhofer.de (F.H.); 3 wbk Institute of Production Science, Karlsruhe Institute of Technology (KIT), Kaiserstr. 12, 76131 Karlsruhe, Germany; volker.schulze@kit.edu

**Keywords:** single screw extrusion, polylactic acid (PLA), surface roughness, tensile properties, Taguchi, fused filament fabrication (FFF)

## Abstract

In this study, the effect of various parameters of a single screw extruder on the rheology and mechanical properties of a polylactic acid (PLA) filament with a 1.75 mm diameter was investigated. The barrel temperature, nozzle and cooling bath temperature, screw speed, nozzle diameter, water bath length, and distance to the nozzle were the process variables. A Taguchi experimental design was implemented using an L8 orthogonal matrix with seven factors and two levels, and their influence on roundness and diameter were evaluated. Among the various processing parameters, the temperature of the cooling bath affected the roundness the most. The mechanical properties and surface roughness of the PLA filament were examined using a tensile test and nanofocus optical system, respectively. Moreover, to assess the filament’s reliability and investigate its behavior further, the filament was used to print 0° plates, and then dog-bone samples were cut from them to evaluate the mechanical properties of the printed specimens. Finally, the results indicate that improved-roundness filaments of 0.004 mm can lead to enhanced mechanical properties in 3D-printed samples with 3.54 MPa.

## 1. Introduction

Fused filament fabrication (FFF) is gaining a great deal of attention due to its low-cost and easy-to-operate machines, high production efficiency, and minimal material wastage [1,2]. A wide range of thermoplastic materials can be used as a filament, which are then extruded through a small nozzle and built up as an object in a melted/softened state based on a layer-by-layer approach [3]. The relevant thermoplastic materials are polylactic acid (PLA) [4,5], nylon [6,7], acrylonitrile butadiene styrene (ABS) [8], polyamide [9], polybutylene adipate terephthalate [10,11], poly(vinyl chloride) (PVC) [12], thermoplastic polyurethane elastomer [12], polypropylene [13,14], polyetheretherketone [15], poly-caprolactone (PCL) [16,17], and polyhydroxybutyrate [18]. PLA exhibits interesting mechanical, optical, thermal, and barrier properties compared to other basic-type plastics [19]. Therefore, among the different basic amorphous thermoplastic materials, PLA is widely used as a raw material in FFF because of its unique characteristics, including excellent mechanical properties, low melting temperature, biodegradability, good thermal stability, good adhesion to the bedplate, and good dimensional accuracy when printing samples [20].

PLA can be manufactured using typical techniques for thermoplastic polymer manufacturing, including extrusion, injection, thermofolding, and blow molding [21]. Extruders are used to produce filament products [22]. The idea of the extruder is simple. From the point of view of the filament manufacturing process, it consists of three steps: hot melt extrusion, cooling, and the winding stage [23]. First, plastics in the form of granules, powders, flakes, or chips are used as the starting material that is fed into the extruder using a top-mounted hopper. Second, the added material is then conveyed forward by friction force that is generated by rotation of the screw and pushed through a die via heating elements. It is then placed over the barrel, where it softens and melts the polymer, converting it to continuous polymer. Finally, a thermocouple is used to control the temperature of the material. The product produced from the die is cooled by air or through a water bath [24]. Usually, extruders are classified into two types: the single screw type and the twin screw type. The single screw is used for polymer-based processing, whereas the twin screw is used for polymer processing with fibers or fillers added and the polymers blended before final molding [25,26]. Owing to the technological process itself, as well as the plasticizing mechanism and its rheological properties, various screw designs can be used depending on the required output product.

The extrusion process is quite well known and understood, but there is still a gap, due to limited studies, in our understanding of the influence of various parameters during the manufacturing process on the filament quality, both for the plastics themselves and those filled with organic fillers (wood flour, cellulose fibers, etc.). Only a few works [22,27,28,29] have focused on filament manufacturing, such as, for example, the study of Park and Fu [27], who presented a review on how the PLA filaments for 3D printing are manufactured. Mishra et al. [28] studied the effect of process parameters on filament diameter in the single screw extrusion of a natural fiber composite. The results revealed that all of the process variables, including nozzle diameter, extrusion speed, and water bath distance, have a significant influence on the filament diameter. Gálvez et al. [29] investigated the influence of the screw speed and plasticizer proportions on the rheological, thermal, mechanical, morphological, and superficial properties of PLA. The study shows that a lower process speed enhances the transitions from viscous to elastic, as well as higher values of the loss modulus, storage modulus, and complex viscosity. Moreover, Fabijanski [22] studied the single screw extrusion process using PLA. It has been noted that PLA behaves very closely to, if not quite the same as, common plastics such as polyethylene (PE) or polypropylene (PP), according to the data available in the literature. Consequently, computer models using obtained data on the rotational speed of the screw, temperature, and other parameters of the PLA extrusion process can help to predict how changes in the process parameters will affect the behavior of the plastic during extrusion, and can also help to determine the optimal parameters to minimize defects in products, increase productivity, and achieve the desired product quality.

To summarize the above-mentioned research work, a variety of thermoplastic materials can be used as a feedstock for FFF. Therefore, there is a general need for custom filament and to widen the understanding of the process. However, most of the current investigations in the literature do not thoroughly clarify the property relationship of different filament fabrication parameters with the roundness, surface roughness, and mechanical properties. Furthermore, most studies in the literature neglect the investigation behind supplemented filaments even though it is also essential to fully understand the effect of the filament process parameters.

This paper attempts to assess the PLA extrusion process, which depends on various process conditions, such as the barrel, nozzle, and cooling bath temperatures; the screw rotational speed, nozzle diameter; water bath length; and the distance to the nozzle. Studying the impact of the previously mentioned extrusion parameters on the filament quality and mechanical properties is important, both from the point of view of the optimization of the production process and the future development of computer models for designing and simulating this process. It should also be noted that granules and filaments from the same manufacturer were used to facilitate the comparison between the commercial filament and the self-manufactured filament.

The paper is organized as follows: First, the experimental approach, including the design of experiment (DoE) plan for various single screw process conditions (e.g., screw speed, barrel and nozzle temperature, nozzle diameter, distance to cooling bath (Cb), Cb length and temperature) for filament production, is presented in Section 2. Then, the printing strategy for 0° unidirectional plates using extruded filament is presented in Section 2.5. The procedure for evaluating the rheology and the thermal and mechanical characterization of the filament, in particular the surface roughness and in-plane tensile properties, is also presented in Section 2.4. Second, the experimental results and analysis are discussed in Section 3. These comprise the roundness, surface roughness, and material characterization based on different extrusion conditions. Additionally, the analysis of variance (ANOVA) that has been performed is shown in Section 3.5. Finally, some meaningful conclusions based on the above analysis are obtained.

## 2. Material and Methods

**Figure 1:**
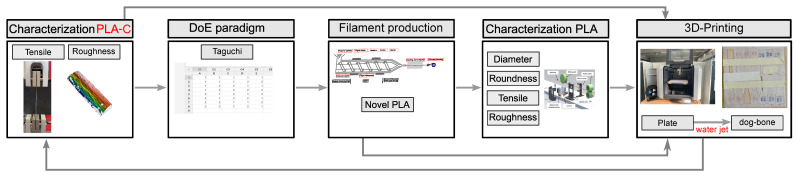
Experimental process flowchart.

The influence of various processing parameters on the rheology and mechanical properties of the PLA filament was explored. Then, the produced filament was used to print 0° unidirectional plates to characterize the mechanical properties compared to samples printed with commercial filament. Thus, this section initially describes the design of experiment (DoE) plan, manufacturing PLA material spools, and the printing samples. Figure 1 illustrates the flowchart of the experimental paradigm proposed in this work. The work started with characterization of the received commercial filament, particularly in terms of tensile and surface roughness. Then, the DoE was applied to help us set level values for filament production. The produced filament roundness and diameter were subsequently measured using a three-axis laser measurement system, followed later by tensile and surface roughness characterization. Finally, the produced filament was used to print plates to validate the reliability of our filaments, including in terms of their tensile characterization. Section 2.3 presents the DoE layout, in which various parameters based on the Taguchi approach are taken into account to produce the filament. In addition, the procedures for characterizing, testing, and evaluating both the produced filaments and printed samples are described thoroughly in Section 2.4 and Section 2.5, respectively.

### 2.1. Material

The type 2003D PLA was supplied in granule form and in a filament spool by a local supplier (Filament2Print [30], Karlsruhe, Germany) and was imported from Ingeo^TM^. This type of material was chosen because it is amorphous, so we could better understand the effect of different extrusion conditions. The granulates datasheet provided us with a processing profile, including melt, feed throat, and nozzle temperatures and screw speed [31,32], which helped us later in selecting our parameters range from two levels (Section 2.3). In addition to the parameter ranges available on the datasheet, the choice for this specific range, as shown in Table 1, was further informed by our own prior knowledge and expertise in this area, ensuring its suitability for producing the desired filament diameter. The two levels were for screw speed (35–50 rpm), extrusion temperature (180–200 °C), nozzle temperature (180–200 °C), nozzle diameter (2–3 mm), distance to cb (5–12 cm), Cb length (25–35 cm), and Cb temperature (24–40 °C).

### 2.2. Manufacturing of the Filament

**Figure 2:**
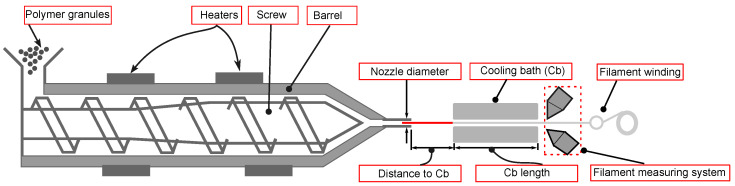
Schematic of single screw extruder.

**Figure 3:**
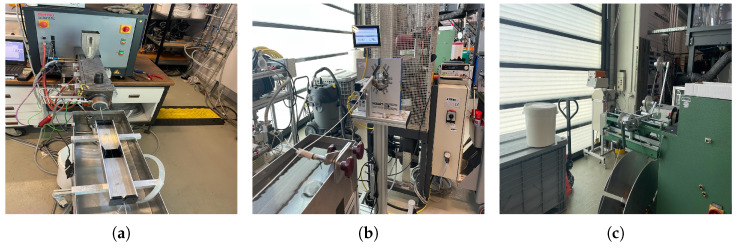
Different parts of the machine used to produce the filament: (**a**) filament extrusion and water cooling bath; (**b**) filament measuring system; (**c**) filament winding.

The PLA filament was fabricated using a single screw extruder. Figure 2 illustrates the schematic of the extrusion process used in this study. The single screw extruder employed in this study, located at the Fraunhofer Institute for Chemical Technology ICT, is illustrated in Figure 3. A detailed view of the extruder, highlighting its various components that were integrated together to manufacture the filament and measure its roundness and diameter is presented in Figure 3. The process started with the extrusion process, then a water cooling bath was attached to cool down the filament (this included a radiator to adjust the water temperature), as presented in Figure 3a. To evaluate the diameter and roundness, a Zumbach diameter scanner and flaw detector in one unit were set up in front of the water cooling bath, as shown in Figure 3b, and, at the end, the winding filament was attached to collect the produced filament and create spool. The diameter and roundness were measured directly as it was extruded from the machine (please see Figure 3c). The 3D laser measurement system used had three axes, allowing for measurements from different directions. The final filament diameter was then calculated as the average of these multiple measurements to ensure the accuracy. To remove the accumulated moisture, the granulates were pre-dried at 60 °C for 6 h and then kept at 40 °C until processing (the timeframe from drying to processing was 1 h to 4 h) before they were used for filament fabrication.

### 2.3. Design of Experiment (DoE)

To reduce the experimental labor cost and time of varying parameters, a process workflow for producing the PLA filament was developed in this study using the Taguchi method. According to the preliminary screening based on some of the literature [33] and the authors’ experience, seven significant factors might affect the PLA filament roundness. Extrusion (screw) speed, cooling bath distance [33], nozzle diameter, and barrel, nozzle, and water cooling bath temperature were selected as control factors in this study. Orthogonal array design L8 [34,35] was generated by referring to the seven factors mentioned earlier with two levels (low and high). The reason for selecting only two levels was that these contain the minimum number of experiments, so it became more effective, reducing experiment time and cost. Table 1 presents the parameters utilized based on the design matrix.

The roundness and surface roughness of the filament and the Young’s modulus for both the filament and the 3D-printed samples with various extruded filaments were chosen as responses to determine the contribution percentage of each factor. The signal-to-noise (S/N) ratio is a quality metric for assessing the impact of input variables towards responses. The roundness and diameter output responses in this investigation are quality characteristics of the `smaller the better’ type. Equation ($\frac{S}{N}=-10\;\log\left(\frac{\sum (Y^{2})}{n}\right)$) is used to estimate the S/N ratios for this characteristic, where Y indicates responses for the given factor-level combination and n refers to the number of responses in the factor-level combination. It is also worth mentioning that the Young’s modulus of the filament and printed samples was assessed with `larger the better’ quality characteristics, as in the following equation: ($\frac{S}{N}=-10\log\frac{\sum_{j}\frac{1}{{Y_{j}}^{2}}}{n}$). Finally, we decided to calculate the overall average within each data group and then calculated the mean of those averages as presented in (Mean of means = $\frac{1}{n}\sum_{i=1}^{m}{\bar{x}}_{i}$), where *m* refers to the number of group data, ${\bar{x}}_{i}$ is the average of the *i*-th group, and *n* is the total number of data points across all groups.

### 2.4. Material Characterization

#### Rheology and Thermal and Mechanical Characterization of Filament

Differential scanning calorimetry (DSC) was conducted using a TA Instrument in a 40 mL/min nitrogen atmosphere. Received commercial samples were weighed prior to the experiment and were approximately 8.49 mg. The heat was increased at a rate of 10 °C per minute and reached a maximum temperature of 210 °C and was then reversed in a cooling phase of 5 °C per minute to reach a final temperature of 25 °C. The obtained data were used to generate a graph of the temperature versus heat flux.

**Figure 4:**
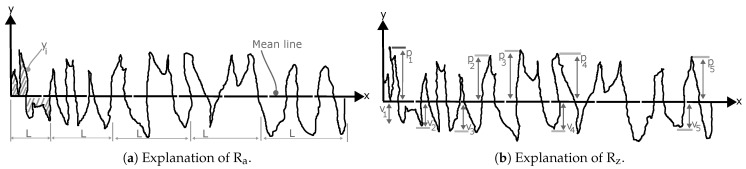
Explanation of R_a_ and R_z_ measurements.

The surface quality of the material depends on the processing conditions [36]. Produced filament might depend on the process parameters, in particular, the screw speed, the temperature of different stages, and the distance from the nozzle to the water cooling bath, which can influence the surface roughness parameters. The surface roughness of a material is basically measured in terms of several parameters, based on ISO 25178 [37], and can be calculated either in two-dimensional (2D) or three-dimensional (3D) forms [38]. In this work, two parameters, namely, the arithmetic average height (also known as the center line average, R_a_) and the ten-point height (R_z_), were chosen to evaluate the surface roughness of the produced filament. The reason for selecting R_a_ is its simplicity, and it considers the most used surface roughness parameters for quality control [38]. However, we chose the R_z_ instead as it is more sensitive to high peaks and deep valleys compared to Ra [38]. Figure 4a and Figure 4b exhibit definitions of the R_a_ and R_z_, respectively, in which p and v refer to the peak and valley, respectively. The mathematical expressions for both $R_{a}$ and $R_{z}$ are presented in the following equations. The *R* values are usually measured in μm. The higher the $R_{a}$ and $R_{z}$ values, the rougher the surface. The expression for the arithmetic average height $R_{a}$ is given by $R_{a}=\frac{1}{l}\int_{0}^{l}\left|yx\right|\;dx$, and the expression for the ten-point height $R_{z}$ is given by $R_{z}=\frac{1}{n}\sum_{i=1}^{n}\left(p_{i}\right)-\sum_{i=1}^{n}\left(v_{i}\right)$, where $p_{i}$ and $v_{i}$ are the profile peaks and valleys, respectively, and *n* represents the number of measurement points.

**Figure 5:**
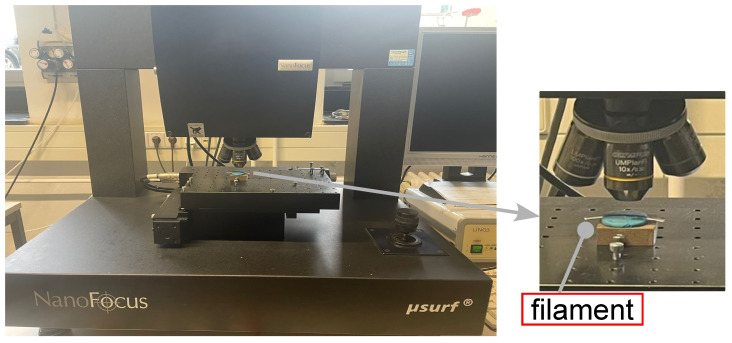
**Left**: procedure for surface roughness measurement by nanofocus; **right**: magnified view of the left figure.

Moreover, the surface roughness of the PLA filament was measured using a nano-optical measurement microscope, as shown in Figure 5. This instrument measures the roughness of a surface. It is commonly used in manufacturing and engineering applications where the texture and surface finish of a material is critical to the functionality of the end product. Similarly, nanofocus optical systems measure surface roughness by using high-resolution confocal microscopy to capture surface topography at the nanometer scale, providing non-contact and high-precision measurements of surface variations [36]. Therefore, roughness was measured along the filament of both sides (see right-hand figure in Figure 5) in order to assess if there were differences regarding their surface finish. R_a_ and R_z_ values were calculated as an average of the values measured at different positions on both sides.

On the other hand, the in-plane tensile properties of the produced filament were tested using a tensile testing machine. There is no standard for testing the filament, so the uniaxial tensile is nearly based on ISO 527-3 [39], which recommends a testing speed of 5 mm/min and load cell of 2.22 kN at room temperature.

### 2.5. Additive Manufacturing

**Figure 6:**
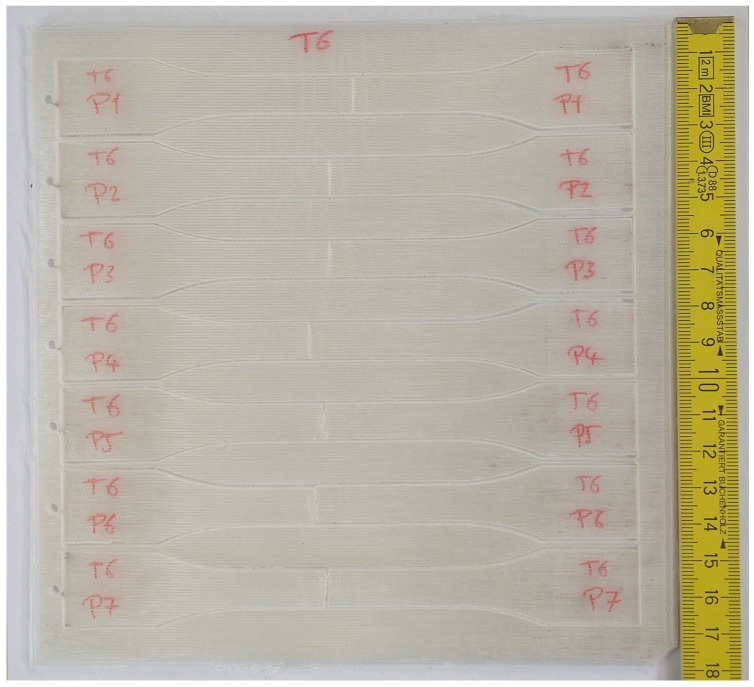
Printed plate used in this study.

The samples were manufactured by means of fused filament fabrication (FFF), with various new filament spools produced. An Anisoprint Composer A3^®^ was used to print the PLA plate specimens with a length and width of 180 mm and thickness of 3 mm, which were subsequently processed into tensile dog-bone specimens by precision cutting using a water jetting machine, as illustrated in Figure 6. The printer consisted of an extrusion head, temperature-control system, building platform, X-Y-Z motion mechanism, etc.

To assess the repeatability, seven specimens of each filament condition were printed at a time. The plate samples were designed based on a prior study, in which it was determined that they should be large enough to capture the anisotropic material properties of fused filament fabrication (FFF) structures [40]. The chosen dimensions of the printed plate enabled thorough testing of the mechanical properties in various orientations, which gave a reliable dataset for the characterization and fit of standard testing equipment [40].

All the tensile specimens were printed adjacent to the bedplate, with the Z-axis serving as the thickness of the samples. Slicer 3D software 5.9.0 was used as a slicing tool to define the printing configurations and toolpath and create the G-code for the printed plate samples. Before printing, the bedplate surface was carefully cleaned and then a special adhesive was applied to allow the specimens to be removed smoothly after printing without harming the samples or bedplate surface. The brim surrounding the part was added to hold its edges. After printing, the printer chamber was left to cool down to room temperature and then the parts were removed from the bedplate. The brim was detached by a cutter, which was also used to smooth the surface. The FFF process parameters used in this work are as follows: 100% solid infill with filling orientation of 0° for all the samples.

#### Mechanical Characterization of Printed Samples

The specimens used for the characterization of the in-plane tensile properties were printed as a plate, and then dog-bone tensile specimens were cut out using a water jet cutting machine to validate the filament properties. The testing facilities (Zwick tensile testing machine) were used with a 2.2 kN load cell. A constant displacement speed of 3 mm/min was applied. The elongation and deformation were calculated using integrated strain gauges in the load cell. The mechanical properties under tension were obtained from graphical data, in which the elastic modules was calculated by the integration of the stress–strain curve from zero to the elastic limit. The maximum percentage of elongation and tensile strength at the break was also obtained.

## 3. Results and Discussion

The main aim of this work was to widen our understanding of the filament manufacturing process, e.g., the effect of process conditions on the filament characteristics in single screw extrusion, and their later influence on the final printed sample’s mechanical properties, using various produced filaments. This was achieved by investigating the effect of various single screw extruder parameters on the structure properties of the produced filaments, including roundness, diameter, surface roughness, and tensile properties. We varied different processing conditions using a Taguchi L8 DoE plan, and their diameter and roundness were analyzed using a 3D laser scanning measurement system. Then, various mechanical properties of both the produced filament and the printed specimens of various configurations were analyzed experimentally using specific test setups with a testing speed of 5 mm/min and load cell of 2.2 kN at room temperature (Zwick tensile testing machine) and supporting analysis software for the surface roughness (µsurf). In this section, several cases comprising different single screw extruder conditions are characterized. Moreover, the results of the mechanical characterization of the manufactured plates are presented.

### 3.1. Thermal Analysis of PLA Filament

**Figure 7:**
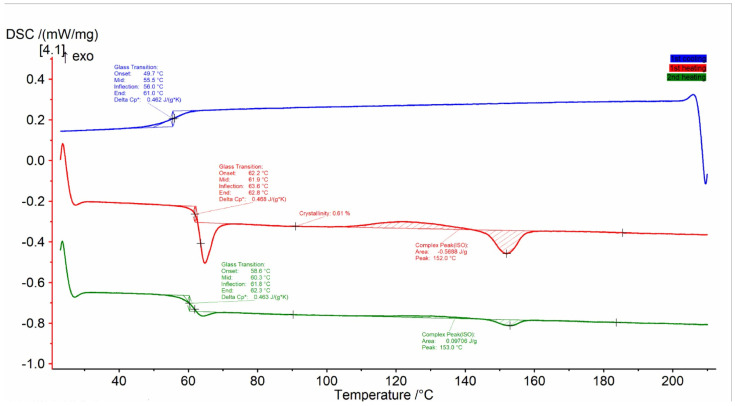
Differential scanning calorimetry (DSC) of PLA commercial filament.

A standard DSC cool-first, heat-second heating method was initially run on a filament sample taken from the received commercial spool, referred to throughout the paper as PLA-C, which was assumed to be amorphous. The DSC result data are shown in Figure 7, which presents the heat flux (mW) versus temperature (°C) for the PLA commercial filament and the extruded filament produced in this study. The spectra show three typical features of amorphous thermoplastics, including PLA, from left to right: heat flux at the glass transition temperature (Tg), an exothermal associated with cold crystallization, and a melting endotherm [41,42]. The T_g_ of the commercial material was 55.5 °C, which is bit lower than the reported values for PLA, while the melting temperature (T_m_) was 153.7 °C, which is within the range of reported values. Differences in these values may be due to additives in the spool material, as well as the extrusion process itself, though this is beyond the scope of the current study. The T_g_ of the commercial and extruded PLA showed a slightly different but almost identical result (that is why we present only one figure of the commercial material as an example). This may indicate that the varying extrusion parameters (to produce PLA) does not affect the nature and distribution of the crystalline phases which form, as well as the capacity for phase changes in the materials above the glass transition temperature.

### 3.2. Surface Roughness of PLA Filament

**Figure 8:**
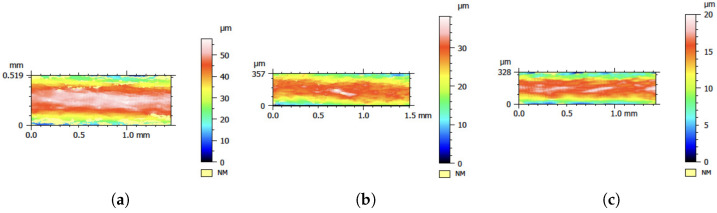
2D surface contour of $R_{a}$ vs. different process variables. (**a**) 2D contour of commercial filament; (**b**) 2D contour of PLA-T2 filament; (**c**) 2D contour of PLA-T4 filament.

The effect of different processing parameters on the surface roughness of the filament was investigated, with a lower surface roughness generally considered to be better. It is usually known that the temperature of the screw and/or nozzle and cooling bath can play a role in developing the surface roughness properties of the filament. Thus, as we mentioned earlier, the surface roughness of the produced and commercial filaments was evaluated at at least six to seven different points or positions along the filament on both sides. Figure 8 shows an example of the 2D contour of one point for the selected produced filament cases. The results reveal variations across different samples, in which the lowest value was achieved by T2 (produced with a screw speed of 35 rpm, barrel and nozzle temperature of 180 °C, nozzle diameter of 3 mm, and distance to cb and cb length of 12 and 35 mm, respectively, and 40 °C cb water temperature), and T4 (produced with the same values as T2, except for a barrel and nozzle temperature of 200 °C and a cb length and temperature of 25 mm and 25 °C, respectively) showed a higher value than T2 and the commercial filament. Despite these measurements, it is important to acknowledge a limitation in the analysis: the surface roughness was captured at specific localized areas rather than in the entire produced filament. This might be why the surface roughness varied significantly across different regions: material heterogeneity and processing conditions. Therefore, the reported values may not fully represent the overall surface characteristics. Further investigations with broader sampling and alternative measurement techniques may be necessary to improve the accuracy and reliability of the roughness assessment.

**Figure 9:**
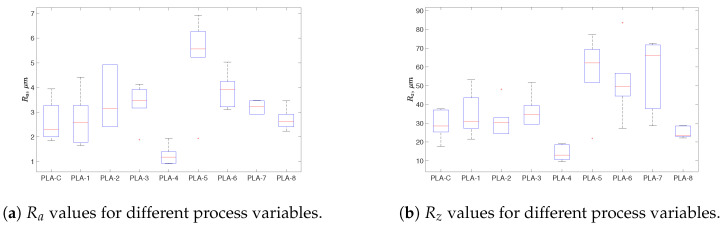
Roughness values for different process variables.

Figure 9 presents the mean values of R_a_ and R_z_, where the values were calculated at at least different six positions along the filament and also within different sides of the filament. Figure 9a presents the mean values for the R_a_ of the extruded filaments compared to the commercial one. Figure 9b shows the R_z_ results of different filaments. Both mean values of R_a_ and R_z_ were calculated at at least different six positions along the filament and also within different sides of the filament. The mean values of R_a_ and R_z_ of the commercial filament were 2.61 µm and 29.11 µm, respectively. The reason for the large differences between R_a_ and R_z_ could be that the filament extruded included high peaks and deep valleys, which agrees with the previous explanation in [38]. On the other hand, the lowest average R_a_ and R_z_ values were 1.260 µm and 13.975 µm, corresponding to PLA-T4, which had a low screw speed, cooling bath length (Cb), and Cb water temperature and a high extrusion temperature, nozzle temperature, and distance from the nozzle to the Cb. The PLA-T4 results were around 51.74% and 52.00% lower for R_a_ and R_z_, respectively, compared to PLA-C, which refers to the commercial filament. The highest R_a_ and R_z_ values of 5.25 µm and 57.45 µm were observed for condition PLA-5, which had a nozzle diameter, extrusion temperature, high screw speed, distance to Cb, temperature of nozzle, and Cb water temperature.

### 3.3. Mechanical Properties of PLA Filament

**Figure 10:**
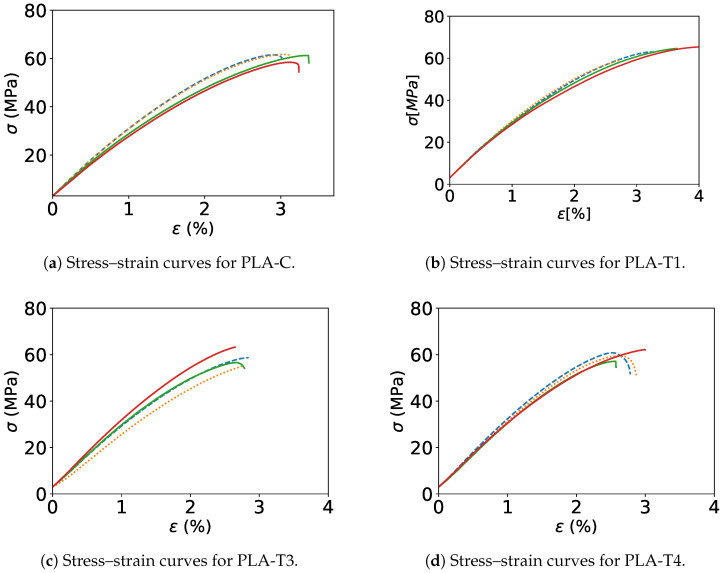
Tensile test results (stress–strain curves) for PLA filaments extruded with different process variables.

Four identical samples were cut from the spool produced and tested for each combined process condition. Overall, 36 samples were tested, including commercial filament. Several cases were selected, in particular, the commercial filament (PLA-C), and both the highest and lowest values. The stress–strain curves for the selected cases are shown in Figure 10.

The highest Young’s modulus with 2.96 GPa was achieved by PLA-T1, as presented in Figure 10b, in which filament was extruded at a low level for all factors. The second highest value of Young’s modulus of 2.96 GPa was achieved by PLA-T4, as illustrated in Figure 10d, which extruded with a mix of high and low factors such as a low screw speed, cb length, and cb water temp and a high nozzle and barrel temperature, nozzle diameter, and distance to cb. The lowest Young’s modulus of 2.51 GPa was observed in PLA-T3 (maximum extrusion and nozzle temperature, cooling bath (Cb) water temperature, and Cb length), as illustrated in Figure 10c. The rest of the Young’s modulus values and the maximum stress for the rest of the cases were within the same range. For example, the difference between the highest Young’s modulus and the lowest for different filament conditions did not exceed 16%. The differences between the highest value for the Young’s modulus and that of the commercial filament (PLA-C) were not more than 4%. The highest maximum stress of 63.03 MPa was observed for PLA-T1 and the lowest of 55.05 MPa for PLA-T6. The difference between the highest and lowest maximum stress (failure stress) was about 12%. It can be observed that the differences between extruded filaments was slightly negligible. Thus, PLA filament mechanical properties are not affected by varying process parameters. A summary of all the Young’s modulus and failure stress values for extruded filament and commercial filament is provided in Table 2.

### 3.4. 3D Printing Samples

A series of tensile tests was performed using the mechanical testing machine mentioned earlier in Section 2.5 at room temperature. The dog-bone-shaped specimens, cut from the printed plates, were subjected to quasi-static uniaxial tensile loading and their stress–strain curves were recorded at a constant crosshead speed of 3 mm/min. It can be observed that failure of the samples occurred at the gauge length of the test coupons. Therefore, the obtained breaking strength refers precisely to the actual breaking strength of the examined samples.

**Figure 11:**
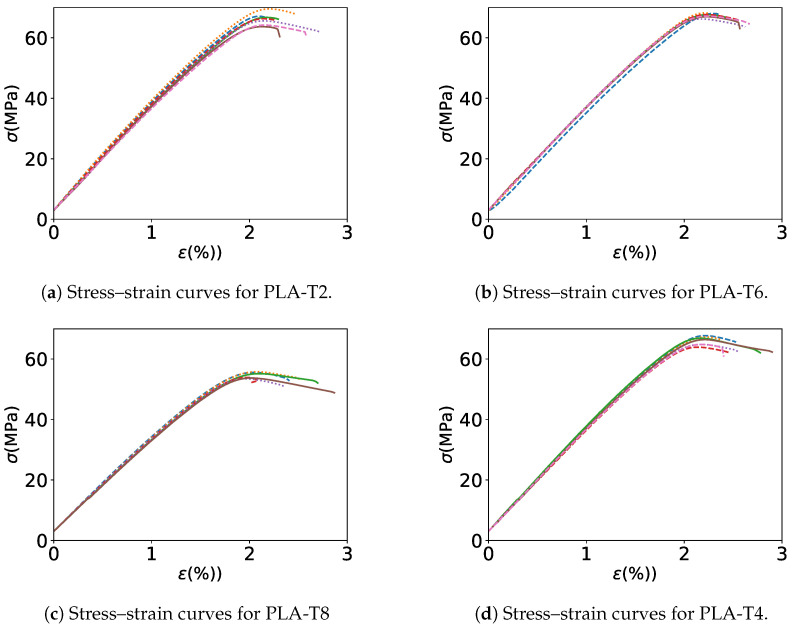
Stress–strain curves of PLA printed plate (cut as dog-bone tensile coupons) manufactured with different PLA filaments.

The results exhibit that the highest Young’s modulus of 3.54 GPa was achieved by PLA-T2, as illustrated in Figure 11a, in which the filament used to print the samples was extruded at low screw speed, extrusion (barrel) temperature, and nozzle diameter and high values for nozzle and Cb water temperature and distance to Cb. Following this, the second highest Young’s modulus of 3.54 GPa was achieved by PLA-T4 (filament produced with high and low levels), as illustrated in Figure 11d. The lowest Young’s modulus of 3.18 GPa was observed in PLA-T8 (maximum screw speed, barrel temperature, nozzle diameter, and cooling bath (Cb) water temperature and low nozzle temperature, Cb length, and distance to Cb), as illustrated in Figure 11b. The highest failure strength of 67.43 MPa was obtained with filament PLA-T6, which used filament spool with low processing factors. The lowest failure strength of 54.61 MPa was obtained with filament PLA-T8, in which filament spool was extruded with low processing parameters such as low nozzle temperature and distance to Cb and high screw speed, barrel temperature, cb water temperature, and nozzle diameter, as shown in Figure 11c.

Unlike with the results for the obtained filament mechanical properties, there was a varying of the results. For example, the difference between the highest Young’s modulus and the lowest for the different filament conditions was around 36%, and the difference between the highest failure strength and the lowest was about 47%. Consequently, printed samples are affected by filament processing conditions. This might again be due to the melting of the filament required to print out the samples. Therefore, the filament processing conditions could be correlated with printed sample characteristics. Despite melting during the printing, still the exact mechanism by which the filament properties influence the printed part is not understood and more studies need to be carried out. A summary of all the Young’s modulus and failure stress values for the printed coupons with several extruded filaments and commercial filaments is provided in Table 3.

### 3.5. Analysis of Variance

Table 4 presents ANOVA results obtained for roundness (R), R_a_, and E for both the filament and final printed samples (plate sample printed with extruded filament). It can be seen that cooling bath water temperature has a significant effect on the roundness and Young’s modulus of the extruded filaments, whereas barrel temperature and nozzle diameter have a major influence on the filament roughness. In addition, cooling bath length has the smallest effect, negligible in the filament roundness and printed samples’ Young’s modulus values. The results show that, in addition to cooling bath water temperature, die diameter has a significant effect on the filament roundness.

**Figure 12:**
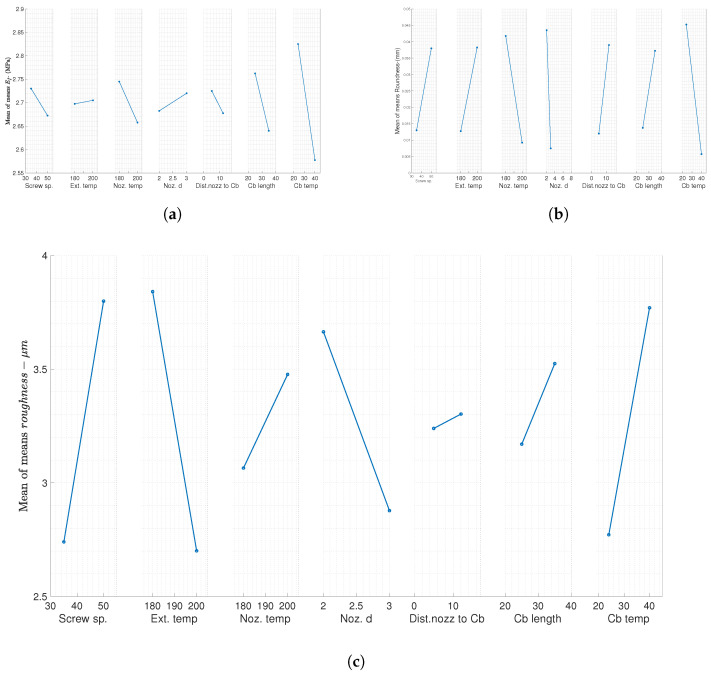
ANOVA analysis: (**a**) Young’s modulus, average of means for filament Young’s modulus; (**b**) filament roundness, average of means for filament roundness; and (**c**) surface roughness, average of means for surface roughness.

Figure 12a,b present the overall means of the Young’s modulus and roundness, respectively, obtained in this study. It can be observed from the results that increasing the cooling water temperature and die diameter improves the roundness of the filament and the Young’s modulus of final printed samples. Moreover, Figure 12c shows the mean of the means of roughness. The results need further studies to confirm and better understand the exact effect on the roughness. The overall means were calculated based on the equation presented earlier in Section 2.3.

Table 5 shows a summary of the different material and mechanical properties achieved in this study, where E_f_ indicates the Young’s modulus results for extruded filament. E_p_ refers to printed plate samples (dog bone cut out from printed plate samples) using different extruded filaments. It can be noted that good mechanical properties are not proportional to low roundness values or surface roughness.

It can be noted that roundness could positively influence the mechanical properties of the printed specimen results. However, roughness values did not show a similar influence. This discrepancy may be due to the fact that roughness was measured only over a small section of the filament, and values can vary across different regions of the spool. Additionally, measuring roughness across the entire spool is practically challenging if not impossible with a nanofocus system. Therefore, further investigation is necessary to fully understand the impact of roughness on mechanical properties.

## 4. Conclusions

This work highlights the employment of Taguchi statistical analysis to investigate single screw extruder process parameters for achieving the lowest roundness of PLA filament. Among various extruder process parameters, the research focused on analyzing the most critical variables, in particular, screw speed, barrel and nozzle temperature, nozzle diameter, distance of cooling bath to nozzle, cooling bath length, and temperature of cooling bath. Additionally, the study used various extruded filaments to print tensile dog-bone samples and analyze their impact on the Young’s modulus.

The following conclusion were drawn from the experimental results:

- Cooling bath temperature (water temperature) and nozzle diameter are the most crucial parameters, with 45% contribution to the filament roundness. For example, low roundness (better rheology properties) was achieved with a high cooling bath water temperature of 40 °C and nozzle diameter of 3 mm, as in PLA-2 and PLA-8.
- Low screw speed, cooling bath length (Cb), and Cb water temperature and high extrusion temperature, nozzle temperature, and distance from nozzle to Cb result in filament with low surface roughness values.
- Varying the single screw extrusion parameters does not markedly influence the Young’s modulus of the extruded filament, in which the percentage between the highest and lowest does not exceed 16.46%, making it a well-suited material for consistent and efficient processing regardless of the processing parameters.
- The mechanical characterization of the produced filament and printed samples shows that there is a correlation between the mechanical properties of the printed parts and the properties of the filament. However, the exact mechanism by which the filament properties influence the printed part, despite melting during the printing process, is unclear and needs to be investigated in future research.
- Despite the considerations mentioned above, we could find the optimum filament condition with low roundness, low surface roughness, and better mechanical properties at a cooling bath water temperature of 40 °C, a nozzle diameter of 3 mm, a distance to cooling bath of 5 cm, a nozzle temperature of 180 °C, a screw speed of 50 rpm, an extrusion temperature of 200 °C, and a cooling bath length of 25 cm.

## Figures and Tables

**Table 1:** Orthogonal array L8 design matrix parameters.

Material Code	Screw Speed	Extrusion (Barrel) Temp.	Nozzle Temp.	Nozzle Diameter	Distance to cb	Cb Length	Cb Water Temp.
	rpm	°C	°C	mm	cm	cm	°C
PLA-T1	35	180	180	2	5	25	24
PLA-T2	35	180	180	3	12	35	40
PLA-T3	35	200	200	2	5	35	40
PLA-T4	35	200	200	3	12	25	24
PLA-T5	50	180	200	2	12	25	40
PLA-T6	50	180	200	3	5	35	24
PLA-T7	50	200	180	2	12	35	24
PLA-T8	50	200	180	3	5	25	40

**Table 2:** Extruded filament mechanical properties.

Sample Name	E-(STD)	$\sigma_{m}$-(STD)
	[GPa]	[MPa]
PLA-$\bar{C}$	2.86 (0.19)	60.71 (1.32)
PLA-$\bar{T1}$	2.96 (0.14)	63.03 (2.47)
PLA-$\bar{T2}$	2.58 (0.20)	55.79 (4.23)
PLA-$\bar{T3}$	2.51 (0.36)	58.34 (3.13)
PLA-$\bar{T4}$	2.87 (0.11)	59.90 (1.86)
PLA-$\bar{T5}$	2.52 (0.46)	61.15 (4.63)
PLA-$\bar{T6}$	2.73 (0.33)	55.57 (7.89)
PLA-$\bar{T7}$	2.74 (0.16)	59.62 (3.02)
PLA-$\bar{T8}$	2.70 (0.23)	59.21 (2.91)

**Table 3:** Printed specimen mechanical properties.

Sample Name	Printed Dog Bone (Cut Out from Plate)		
	$\sigma_{m}$-[MPa]	$E_{m}$ [GPa]	$\rho_{m}$ [g/cm^3^]
$\bar{T1}$	63.78 ± 2.22	3.38 ± 0.13	1.15 ± 0.02
$\bar{T2}$	66.18 ± 1.81	3.54 ± 0.18	1.15 ± 0.01
$\bar{T3}$	65.59 ± 1.49	3.45 ± 0.06	1.16 ± 0.02
$\bar{T4}$	65.98 ± 1.34	3.46 ± 0.10	1.13 ± 0.02
$\bar{T5}$	56.96 ± 3.23	3.20 ± 0.12	1.04 ± 0.02
$\bar{T6}$	67.43 ± 0.63	3.42 ± 0.16	1.12 ± 0.02
$\bar{T7}$	63.45 ± 0.61	3.52 ± 0.20	1.10 ± 0.00
$\bar{T8}$	54.61 ± 0.92	3.18 ± 0.03	1.07 ± 0.02

**Table 4:** ANOVA of roundness, roughness (R_a_), and Young’s modulus (E).

	Screw Speed	Barrel Temp.	Die Temp.	Die Diameter	Distance to Cb	Cb Length	Cb Water Temp.
Roundness of the extruded filament							
Contribution%	9.66	10.05	16.32	20.03	11.26	8.53	24.11
R_a_ of the extruded filament							
Contribution%	25.18	29.06	3.79	13.83	0.08	0.57	22.28
E of the extruded filament							
Contribution%	3.63	0.06	8.41	1.54	2.48	16.50	67.35
E of the printed plate samples							
Contribution%	24.26	2.94	3.67	2.20	13.23	33.82	19.85

**Table 5:** Material and mechanical properties of the different filament and printed plate produced.

Material Code	Roundness (STD)	R_a_ (STD)	E_f_ (STD)	E_p_ (STD)
	[mm]	[µm]	[GPa]	
PLA-C	0.006 (0.002)	2.611 (0.834)	2.86 (0.19)	–
PLA-T1	0.029 (0.002)	2.713 (0.434)	2.96 (0.14)	3.38 (0.13)
PLA-T2	0.004 (0.000)	3.496 (0.655)	2.58 (0.20)	3.54 (0.18)
PLA-T3	0.006 (0.003)	3.491 (0.468)	2.51 (0.36)	3.45 (0.06)
PLA-T4	0.013 (0.005)	1.260 (0.331)	2.87 (0.11)	3.46 (0.10)
PLA-T5	0.009 (0.000)	5.250 (1.460)	2.52 (0.46)	3.20 (0.12)
PLA-T6	0.009 (0.002)	3.908 (0.635)	2.73 (0.33)	3.42 (0.16)
PLA-T7	0.013 (0.004)	3.205 (0.137)	2.74 (0.16)	3.52 (0.20)
PLA-T8	0.004 (0.000)	2.846 (0.307)	2.70 (0.23)	3.18 (0.03)

## Data Availability

All data generated or analyzed during this study are available from the corresponding author upon reasonable request.
